# Parents', Families', Communities' and Healthcare Professionals' Experiences of Care Following Neonatal Death in Healthcare Facilities in LMICs: A Systematic Review and Meta‐Ethnography

**DOI:** 10.1111/1471-0528.17982

**Published:** 2024-10-18

**Authors:** Charlotte Wilson, Bethany Atkins, Richard Molyneux, Claire Storey, Hannah Blencowe

**Affiliations:** ^1^ Centre for Maternal Child and Reproductive Health (MARCH) London School of Hygiene and Tropical Medicine London UK; ^2^ University Hospitals Plymouth NHS Trust Plymouth UK; ^3^ Institute for Women's Health University College London London UK; ^4^ Stillbirth Advocacy Working Group, International Stillbirth Alliance New York, NY USA

**Keywords:** bereavement care, healthcare setting, hospital, LMIC, neonatal mortality

## Abstract

**Background:**

Ninety‐eight percent of neonatal deaths worldwide occur in low‐ and middle‐income countries (LMICs), yet there is little bereavement care guidance available for these settings.

**Objectives:**

To explore parents', families' and healthcare professionals' experiences of care after neonatal death in healthcare facilities in LMICs.

**Search Strategy and Selection Criteria:**

Four databases were searched for peer‐reviewed literature, meeting the inclusion criteria of qualitative studies exploring the experiences of people who provided or received bereavement care following neonatal death in a LMIC healthcare setting.

**Data Collection and Analysis:**

Data were collected by two independent reviewers, collated through line‐by‐line coding and then reciprocal and refutational translation, and analysed through Noblit and Hare's seven‐step meta‐ethnography approach to create first‐, second‐ and third‐order themes.

**Main Results:**

Seven first‐order themes extracted from the literature included emotional responses, social relationships, staff and systems, religion, connecting with the baby, coping strategies and economic concerns. From these data, three third‐order themes arose: The individual, the healthcare setting and the community/context.

**Conclusions:**

Overarching themes in bereavement care shape grief responses and are often similar across geographical locations. Analysing these similarities allows a deeper understanding of the important elements of bereavement care and may be helpful to inform the creation of high‐quality, bereavement care guidelines suitable for use in LMIC settings.

## 1 Introduction

Oh God, please help me, I need someone to help me pass through this pain. [1]

The loss of a baby is a traumatic experience felt deeply by all involved. Globally, 2.3 million neonates die annually in their first month of life, with around 98% of these deaths occurring in low‐ and middle‐income countries (LMICs) [2]. This impacts the emotional, social and physical well‐being of parents, families, communities and healthcare professionals [3]. In these times of grief, it is vital that healthcare professionals and community leaders have clear guidance to help them navigate the grieving process alongside families. This guidance should be specific to neonatal mortality and based around local knowledge and culture, something which is not currently available in many locations [4].

This systematic review is the first analysis of literature surrounding experiences of care after a neonatal death in LMIC healthcare facilities, which considers the perspectives of healthcare professionals, communities, parents and families. This is necessary as current guidance is based around results from high‐income countries (HICs) or data on stillbirths [4, 5, 6], which may not be fully generalisable to these contexts [4, 5, 6]. As 80% of births globally now occur in healthcare facilities, this review focuses on healthcare settings from many perspectives, to give a broad understanding of this topic [7]. This is important for obstetric teams to be aware of, as more than a third of neonatal deaths occur on day 1 postpartum and 75% within 7 days, most frequently to women whose babies born preterm or with other complications and commonly while they remain under obstetric care [8]. This information may then be used to aid the creation of targeted interventions and guidance, including towards improving obstetric supportive engagement in subsequent pregnancies, in settings where it is most needed.

## 2 Methods

We undertook a systematic review and meta‐ethnography of parents', families' and healthcare professionals' experiences of care after neonatal death in healthcare facilities in LMICs. The review is registered with PROSPERO (reference: CRD42022345072), and we report our findings in accordance with PRISMA and eMERGe guidance (Appendix S1) [9, 10].

MEDLINE, EMBASE, PsychInfo and Global Health databases were searched on the 24 June 2022 including terms related to ‘experience of care’, ‘neonatal death’, ‘healthcare facilities’ and ‘low‐ and middle‐income countries’, with no date limits applied (Appendix S2).

Screening and data extraction were completed by two independent reviewers. Studies were included if they provided qualitative data on parents', families' or healthcare professionals' experiences of neonatal death (defined as death during the first 28 days of life following a live birth) in healthcare facilities (defined as any hospital or healthcare centre) in any country classified by the World Bank as a LMIC [11]. Where studies included both stillbirth and neonatal mortality, these were separated where possible, and a note on affected studies was made available (Appendix S4). The study quality and risk of bias were assessed using the Joanna Briggs Institute (JBI) critical appraisal tools (Appendix S4) [12].

The data were analysed using Noblit and Hare's seven‐phase meta‐ethnography approach [13]. First, a ‘long list’ of first‐order themes was extracted iteratively from participant‐generated data from the included studies, using thematic analysis techniques of line‐by‐line coding [14]. This ‘long list’ was compared across studies through constant comparison [15]. Through both reciprocal and refutational translation, these themes were grouped into broader categories to create a ‘short list’ of fewer than 10 themes. This short list was divided into ‘major’ and ‘minor’ themes. Major themes were those arising in more than three‐quarters of studies, and minor themes were those arising in fewer than three‐quarters of studies, contributing less to the line of argument synthesis (Appendix S3).

Next, second‐order themes, arising from the interpretations and analysis of individual study authors, were extracted separately by reviewing the authors' subheadings, conclusions and key arguments. Finally, third‐order themes were generated by combining all elements of the experience of bereavement care described in the first‐ or second‐order themes in a single model, based on the structure of the socioecological model for public health [16].

## 3 Results

**FIGURE 1:**
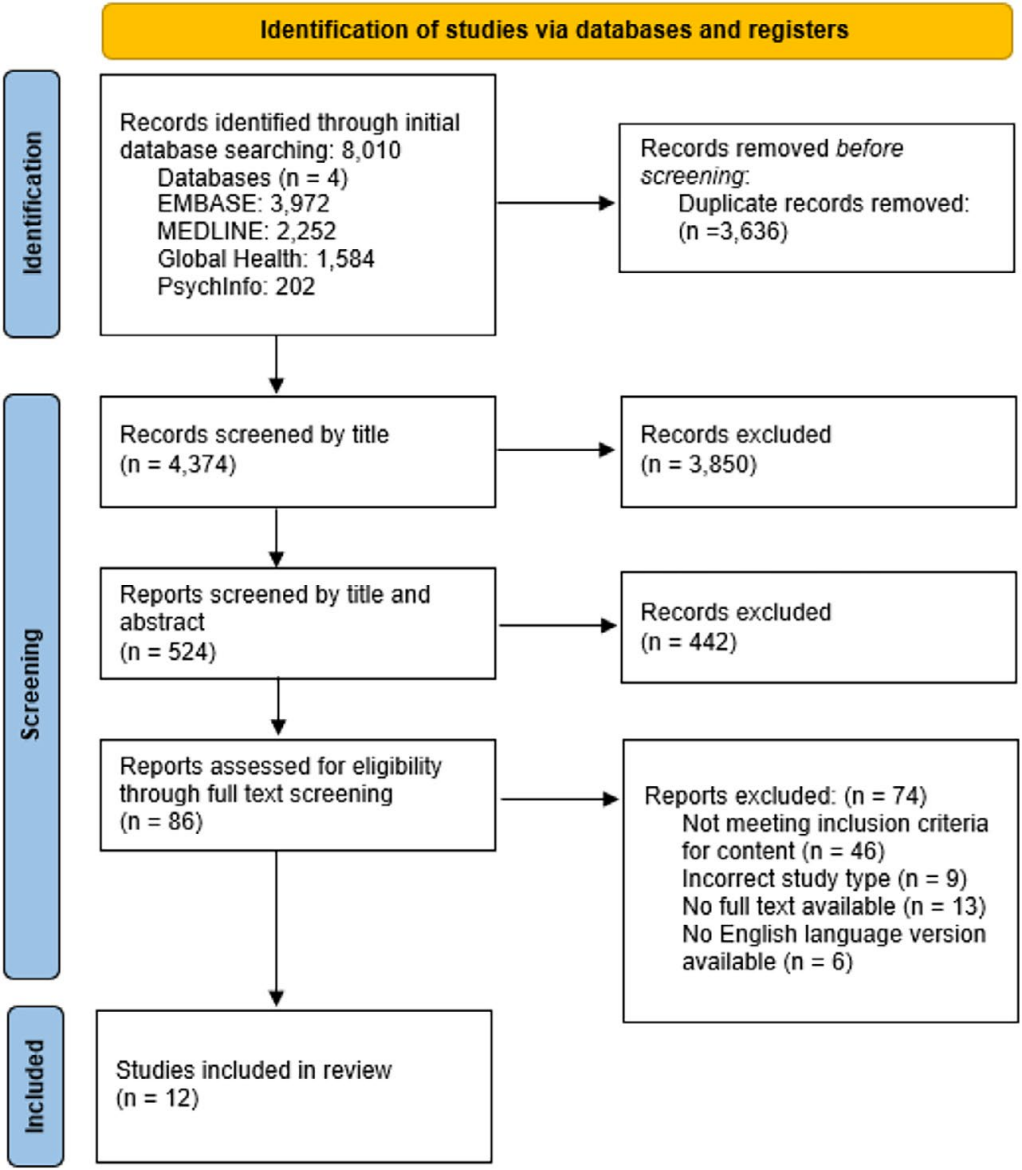
PRISMA diagram showing the study selection process. In total, 141 participants or households that experienced neonatal mortality are included in this review. In five of the included studies [1, 18, 24, 26, 28], it wasn't possible to fully distinguish between the testimonials of mothers experiencing stillbirth versus neonatal mortality, meaning the experiences of up to 161 participants may be included.

**FIGURE 2:**
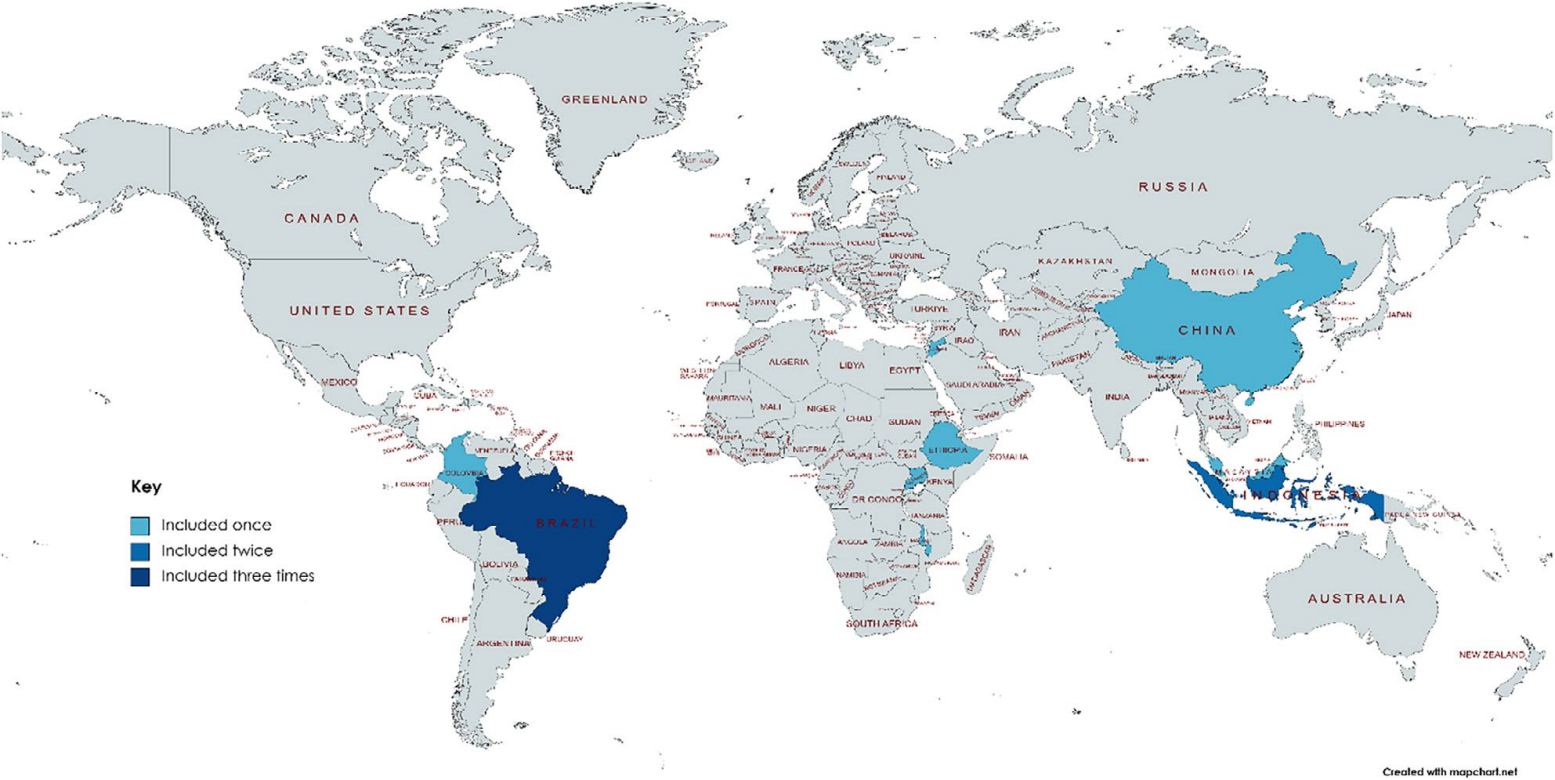
Map showing the location of included countries.

A total of 12 qualitative studies from nine LMICs, published between 2004 and 2022, were included (Figures 1 and 2). Ten studies focus on the experience of parents, one explores the experiences of nurses and one focuses on midwifery students. The studies were of moderate‐to‐high quality with moderate‐to‐low risk of bias (Appendix S4).

### 3.1 First‐Order Themes

Forty‐nine ‘long list’ first‐order themes were identified. These were condensed into four major short‐list themes present in more than three‐quarters of papers and three minor themes (Table 1). No studies were found to refute the conclusions of another.

**TABLE 1:** Occurrence of first‐order themes across included studies.

Key	Theme discussed	Theme not discussed

	Included studies											
Location	Malaysia	Brazil	Malawi	Indonesia	Ethiopia	Brazil	Brazil	Jordan	Indonesia	Columbia	Uganda	China
References	[1]	[16]	[17]	[18]	[19]	[20]	[21]	[22]	[23]	[24]	[25]	[26]
(i) Emotional response												
(ii) Social relationships												
(iii) Staff and systems												
(iv) Religion												
(v) Connecting with the baby												
(vi) Coping strategies												
(vii) Economic concerns												

#### 3.1.1 Major Theme 1: Emotional Response

In all studies, participants described emotional responses to neonatal death as vital to the grieving process. The most frequently mentioned were sadness, grief and guilt, present in eight studies. Some parents exhibited immediate expressions of grief, while some felt ‘empty’ or experienced a feeling of ‘sorrow’ later, perhaps after leaving the hospital. Parents also frequently described anticipation or excitement, which morphed into grief following the death. This was supported by nurses, who described a vital aspect of care being knowing whether to give parents space or to become involved, as all grief responses were very individual [17].

I was filled with sorrow because I was expecting something, I was eagerly waiting, and I was also happy that I would have a baby. I was heart‐broken… [18]

A feeling of guilt was reported across countries. Notably, mothers experienced more guilt in settings where they were unsure of the cause of death, as this uncertainty sometimes led them to blame themselves.

My feeling of guilt is certainly greater than my feeling of sadness. I kept asking myself how come I was so negligent. [19]

Other common emotions were confusion, isolation, loneliness, emptiness and anger. These generally decreased over time, but triggers such as seeing other children, hearing babies crying and discussing their baby's death could lead to the re‐occurrence of strong feelings at any time. In three studies, women reported fear surrounding future pregnancies and wanted reassurance that the death would not recur.

#### 3.1.2 Major Theme 2: Social Relationships

The role of family and friends in the grieving process was described in 11 studies. Participants received emotional support from partners, family, close friends, colleagues and the wider community. This was often positive, with people receiving comfort, support and sharing their grief with others, particularly with husbands and parents. In a study of nurses, they believed that admitting family to the ward to grieve with the parents was good practice and generally helpful [17].

Conversely, some bereaved parents reported being unable to share the full experience of grief, either because they were trying to appear ‘strong’ or because they felt that others didn't understand their pain. In other instances, the family withheld the news of the death from the mother. Both of these impacted social relationships. There were also instances of communities understating the importance of a newborn's death, with parents feeling a loss that the community did not acknowledge. This was particularly centred around the concept that a newborn is ‘not yet a person’, or that they can be ‘replaced’ by other children.

It was only a neonate so no one was set to mourn. [20]

#### 3.1.3 Major Theme 3: Staff and Systems

This theme included the role of staff, as well as hospital organisation and layout. Eleven studies included areas for improvement, particularly staff training, as it was often reported that staff did not know what to do following a neonatal death and sometimes avoided speaking to bereaved parents. This led to feelings of abandonment; feeling that staff ignored the parents to focus on the baby, or ignored the father to focus on the mother; and some parents blamed staff negligence for their baby's death.

There is nothing that they were doing, when the child died, they were just walking about. [18]

Across contexts, mothers wanted more information surrounding the cause of death and the support available. Furthermore, in two studies, there were concerns regarding ward organisation, with participants watching multiple babies die in the same bed in Ethiopia, and bereaved mothers moved to a ward with living babies in Malaysia [1, 20]. Both situations were reported as profoundly distressing. In Indonesia, a woman reported being forced to leave the hospital immediately after the death, contributing to the overall difficulty of the experience [19].

Conversely, care from ward staff and nurses was appreciated in all settings, with most studies reporting emotional support from staff as very important. Additionally, the value of counselling or a psychologist in the weeks after the death was raised in three studies. In one study, physical care was viewed as most important, but, while this observation was never refuted, it was not replicated elsewhere [21].

#### 3.1.4 Major Theme 4: Religion

Nine studies discussed religion. Generally, religious beliefs offered comfort to parents and were used by friends, family and staff to help rationalise death. In a Malaysian study of bereaved Muslim women, religion was reported as a central theme and an important method of rationalising what had happened as well as helping parents to manage their grief [1].

My family and friends always remind me to be patient and take this as a test from God… I hope Allah will bless me and solve it [the grief] in the near future. [1]

Religious ceremonies and practices were commonly carried out following the baby's death, providing the parents with support and a sense of providing for their child. In one study, it was reported that some participants questioned their religious beliefs following bereavement [22], but this was not translated across studies.

#### 3.1.5 Minor Theme 1: Connecting With the Baby

Connecting with the baby was identified as a theme in six studies. Opportunities to connect with the neonate were considered important by nurses, who reported allowing mothers to bathe, clothe and change their babies after the death [17]. One nurse described this as an ‘end to the story’ and a way of providing closure [17]. Women in Jordan also disclosed that being denied the opportunity to connect with their baby was detrimental to their grieving process and a source of later regret [23].

I wish I saw him before burial. I feel very sad that I didn't see him and hold him. [23]

#### 3.1.6 Minor Theme 2: Coping Strategies

Four studies discussed coping strategies used by parents to help manage grief [17, 19, 24, 25]. These included keeping busy with other children, taking pleasure from a new pregnancy, or sharing stories.

The first day after the baby was buried, I thought ‘OK, maybe I should focus on my other children.’ I think that was my destiny, I should receive it and look at the bright side. [24]

#### 3.1.7 Minor Theme 3: Economic Concerns

Studies in Uganda, Ethiopia and Brazil noted that the cost of hospital treatment was a barrier to service use, particularly as neonates are not yet productive family members [20, 21, 26]. This sometimes meant that families couldn't afford extended hospital care, or that bereavement experiences were worsened by needing to work additional hours following the death.

After the loss, I wasn't at peace because I kept paying debts yet the baby was not there. [26]

### 3.2 Second‐Order Themes

Twenty‐five second‐order themes were extracted, two to five from each of the included studies. These reflect the views of individual study authors and were mapped to the first‐order themes generated above. No new themes emerged at this stage of the analysis.

### 3.3 Third‐Order Themes

**FIGURE 3:**
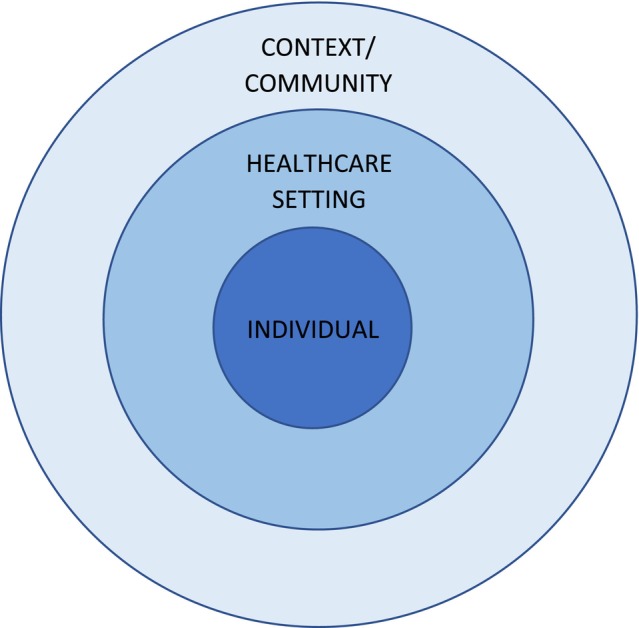
Model displaying the third‐order conceptualisations of bereavement care in hospitals in LMICs.

Three third‐order themes were generated from the first‐order concepts, combining all elements of the experience of bereavement care in a single model. These themes can be conceptualised as linking together, with the individual always at the centre of care (Figure 3). Surrounding each individual is the healthcare setting in which they experience bereavement, and these all take place within the context of their community, shaping attitudes and practices in a more distal, but vital, manner.

#### 3.3.1 Final Theme 1: The Individual

A single neonatal death affects many people, and each should be considered when providing care. The most discussed individuals were mothers; however, fathers' viewpoints were highlighted in two studies [20, 25]. A recent systematic review assessing men's responses to perinatal bereavement suggests that paternal grief is an important element that is often overlooked [27].

The individual can also be viewed as an ‘individual unit’, for example, both parents, the household or the entire family, all of whom are affected by the death, and are discussed as participants in the grieving process. Other affected individuals would include hospital staff, who may become very involved and will require coping strategies to manage this [17, 27].

Individual's response to bereavement was highly diverse, encompassing a broad spectrum of emotions. This was considered normal, with a consensus that people should be supported to grieve in the way which feels most natural to them. This same principle extends to coping strategies, recognising that various methods of coping are effective for different people. These differences may be linked to personality, experiences, religion or cultural norms, contributing to a highly variable and individualised approach to grieving and coping [28].

This is not to say that there were no commonalities. Emotional responses were consistently reported across all studies, and being allowed to spend time with the baby after death was reported as helpful in multiple locations. Indeed, in settings where this connection was not possible, parents reported more regret and late‐night waking. Therefore, overarching principles such as experiencing an emotional response and benefitting from connecting with the baby can likely be generalised widely, while the exact ways in which these occur will be more specific to individuals and contexts.

#### 3.3.2 Final Theme 2: The Healthcare Setting

Experiences of bereavement care varied not just between individuals but between settings. There were no specific differences noted between private and public, or middle‐income and low‐income settings, although certain system‐related issues were more prevalent in individual studies. There were, however, consistent principles for good care across contexts. Well‐trained and supportive staff of adequate numbers to support both physical and emotional care were valued, while the grieving process was helped by a clear explanation of the causes of death and how to access support.

Structural elements were also important. Allowing bereaved parents to have separate spaces where they would not be disturbed was beneficial, with locations where this was not possible highlighting distress on hearing babies crying or seeing other babies die. There were also mentions of economic concerns, highlighting that parents will only access services if they can afford to, and concerns about costs may add stress to an already difficult experience.

#### 3.3.3 Final Theme 3: Context and Community

Local practices, attitudes, religion and beliefs surrounding death and the role of the neonate shaped experiences. In some studies, community influence was beneficial, with support from close family and friends often being helpful. Some community attitudes were damaging, however, with a dismissal of neonatal death as unimportant due to the age of the deceased posing a challenge for parents, who felt the death of their baby was more important than their community believed. Therefore, context and community were vital elements of bereavement care practices which should be considered when attempting to generalise findings.

## 4 Discussion

### 4.1 Main Findings

This review demonstrates that neonatal mortality impacts, and is affected by, the individuals involved, the healthcare setting they operate within and the context of their local community. Specifically, the themes arising from the literature were an emotional response, social relationships, staff and systems, religion, connecting with the baby, coping strategies and economic concerns. These all shaped the experience of bereavement care following neonatal mortality in LMICs and should be considered when developing any future guidance.

### 4.2 Interpretation With Comparison to Wider Bereavement Care Literature

There is a paucity of research discussing bereavement care following neonatal mortality in LMIC healthcare settings. Stillbirth literature suggests that bereaved parents can experience long‐term effects including physical and psychological consequences and tangible and intangible costs [29]. Optimal supportive bereavement care from the time of death onwards for affected families can play an important role in reducing many of these consequences. While research specifically focusing on neonatal death is limited, studies on perinatal death have revealed a threefold increased risk of postnatal depression [30]. Neonatal death within the first week of life, often prior to hospital discharge or completion of cultural acceptance rituals such as naming ceremonies, may have a similar impact as stillbirth. Comparisons can be made with the existing literature on care after perinatal mortality, or in high‐income settings, to assess the generalisability of the themes arising in this review. A 2020 systematic review of perinatal and child mortality included studies covering neonatal mortality from Ghana, South Africa and the Central African Republic [4]. It concluded that some expressions of grief are universal but affected by cultural differences, with Ghanaian mothers expressing a stronger desire for connection with their neonates than women in other settings. This supports results from this analysis; however, the detail included about these studies is limited, and the conclusions are narrow.

A 2019 systematic review by Shakespeare et al., including 34 studies on the experience of care following stillbirth in LMICs, found the most frequent thematic sentences related to ‘positive community support’, ‘women's experiences of grief with multiple manifestations that may go unrecognised’ and ‘awareness of and support for appropriate coping mechanisms’ [31]. These are strongly correlated with the conclusions drawn in this study, particularly the first‐order categories of social relationships, emotional responses and coping strategies.

One key difference between Shakespeare's stillbirth review and this study was around the discussion of stigma. While stigma was a key theme in stillbirth data, it did not arise in the literature surrounding neonatal mortality. It is possible that the difference in the timing of death may affect the levels of stigma experienced. This is an important question which may merit further investigation, as stigma is known to impact health outcomes [32].

A 2022 paper reviewed clinical practice guidelines from high‐income countries on perinatal bereavement care [33]. It identified two ‘highest quality’ guidelines, of which only one covered neonatal mortality. This guideline was produced by the Perinatal Society of Australia and New Zealand (PSANZ) [34]. PSANZ's recommendations for respectful and supportive care, found in section three, support major themes uncovered in this review [34]. They advocate for good communication, allowing connection with the baby, keeping parents informed and being prepared for a range of emotional responses. Additionally, PSANZ discusses the importance of ‘space and surroundings’, linking to the third‐order theme of ‘healthcare settings’ [34].

There were, however, differences between PSANZ guidance and this research. Some interventions, such as autopsies and extensive testing, may not be feasible in settings with fewer resources. Furthermore, obtaining mementos with the baby is discussed extensively in PSANZ guidance but was not discussed in any studies in this review and may not always be culturally appropriate. Therefore, this concept warrants further investigation before any guidance is produced, and a ‘bottom‐up’ approach to understanding local bereavement practices should be used to ensure that guidance is fully applicable to the setting in which it is intended for use.

### 4.3 Strengths and Limitations

This research is based on the interpretivist paradigm, seeking subjective knowledge and assuming that reality can be understood through social constructions such as consciousness, language and shared meanings [35]. The lead researcher is a UK‐based, white female with a background in clinical medicine. This researcher has most experience in health care in HICs, spending only short periods in LMICs. Therefore, despite methodological efforts to limit the impact of bias including the use of multiple reviewers, following structured guidelines and using JBI checklists, it is likely that interpretations presented in this research will be influenced by these intersecting elements.

A limitation of this paper is that only 12 studies, from nine separate countries, were identified. While this duplication may not be a major concern, as countries are large, socioeconomically and culturally variable, the lack of studies reflects a paucity of research and reduces generalisability. However, data saturation was reached, and the conclusions overlapped with other, comparable literature [4, 5, 31, 34, 36]. It is essential, however, that local social, cultural and economic factors are considered before principles are applied in other settings.

Another limitation was the lack of participant variety, as most studies focused on parents' experiences of care, meaning groups such as staff and extended families are under‐represented. There were also two studies in which it was not fully possible to separate data on stillbirths from neonatal mortality, meaning some participants experiencing stillbirth may have been included. Furthermore, by only including healthcare settings, bias is introduced towards people who can access care [37]. This may affect themes such as ‘economic concerns’, making them appear less prevalent, as opinions of those who cannot afford care will not be included. To reduce this impact, ‘minor themes’ were included in the main text alongside ‘major themes’, to allow for the fact that they may represent a wider body of opinion than the literature demonstrated. It is, however, recommended that this report is combined with other, wider research and expert opinion with the aim of contributing to the development of appropriate bereavement care guidance for LMICs.

## 5 Conclusions

In conclusion, this review demonstrates that overarching themes in bereavement care link to the individual, the healthcare setting and the context in which the death occurs. Each of these factors intersects with the others, and thus no two bereavement experiences are the same. It is, however, possible to extract similarities between experiences to develop a deeper understanding of how bereavement care may be optimised to support people through the grieving process. Further research should focus on more localised data generation and analyses to ensure that any recommendations made are locally applicable and culturally appropriate, ensuring that all bereaved people are provided with the highest possible standard of care.

## Author Contributions

The study was conceptualised and designed by CW, BA, HB and CS. CW and RM undertook the review and data extraction. CW led the analysis and drafted the initial draft of the paper with inputs from all authors. All authors helped revise the manuscript. All authors reviewed and agreed on the final version.

## Conflicts of Interest Statement

None declared.

## Supporting information


Appendix S1.

## Data Availability

The authors have nothing to report.
