# Supplementary material for: Parents', Families', Communities' and Healthcare Professionals' Experiences of Care Following Neonatal Death in Healthcare Facilities in LMICs: A Systematic Review and Meta‐Ethnography
Source: BJOG. 2024 Oct 18;132(3):346–54. doi: 10.1111/1471-0528.17982 (PMC11704079; doi:10.1111/1471-0528.17982)
Supplement: Supplementary file 1 — Appendix S1. [file BJO-132-346-s001.docx]

**Running title: Experiences of care following neonatal death in LMICs**

**Appendix**

Contents

[**Appendix S1** PRISMA reporting guidance for systematic reviews. 2](#_Toc176794163)

[**Appendix S2:** Example of the detailed search strategy used for OVID medline. 6](#_Toc176794164)

[**Appendix S3:** Table describing first order themes extracted from each study. 8](#_Toc176794165)

[**Appendix S4:** Detailed description of included studies. 21](#_Toc176794166)

# **Appendix S1** PRISMA reporting guidance for systematic reviews.

| **Section and Topic** | **Item #** | **Checklist item** | **Location where item is reported** |
| --- | --- | --- | --- |
| **TITLE** | | |  |
| Title | 1 | Identify the report as a systematic review. |  |
| **ABSTRACT** | | |  |
| Abstract | 2 | See the PRISMA 2020 for Abstracts checklist. |  |
| **INTRODUCTION** | | |  |
| Rationale | 3 | Describe the rationale for the review in the context of existing knowledge. |  |
| Objectives | 4 | Provide an explicit statement of the objective(s) or question(s) the review addresses. |  |
| **METHODS** | | |  |
| Eligibility criteria | 5 | Specify the inclusion and exclusion criteria for the review and how studies were grouped for the syntheses. |  |
| Information sources | 6 | Specify all databases, registers, websites, organisations, reference lists and other sources searched or consulted to identify studies. Specify the date when each source was last searched or consulted. |  |
| Search strategy | 7 | Present the full search strategies for all databases, registers and websites, including any filters and limits used. |  |
| Selection process | 8 | Specify the methods used to decide whether a study met the inclusion criteria of the review, including how many reviewers screened each record and each report retrieved, whether they worked independently, and if applicable, details of automation tools used in the process. |  |
| Data collection process | 9 | Specify the methods used to collect data from reports, including how many reviewers collected data from each report, whether they worked independently, any processes for obtaining or confirming data from study investigators, and if applicable, details of automation tools used in the process. |  |
| Data items | 10a | List and define all outcomes for which data were sought. Specify whether all results that were compatible with each outcome domain in each study were sought (e.g. for all measures, time points, analyses), and if not, the methods used to decide which results to collect. |  |
|  | 10b | List and define all other variables for which data were sought (e.g. participant and intervention characteristics, funding sources). Describe any assumptions made about any missing or unclear information. |  |
| Study risk of bias assessment | 11 | Specify the methods used to assess risk of bias in the included studies, including details of the tool(s) used, how many reviewers assessed each study and whether they worked independently, and if applicable, details of automation tools used in the process. |  |
| Effect measures | 12 | Specify for each outcome the effect measure(s) (e.g. risk ratio, mean difference) used in the synthesis or presentation of results. |  |
| Synthesis methods | 13a | Describe the processes used to decide which studies were eligible for each synthesis (e.g. tabulating the study intervention characteristics and comparing against the planned groups for each synthesis (item #5)). |  |
|  | 13b | Describe any methods required to prepare the data for presentation or synthesis, such as handling of missing summary statistics, or data conversions. |  |
|  | 13c | Describe any methods used to tabulate or visually display results of individual studies and syntheses. |  |
|  | 13d | Describe any methods used to synthesize results and provide a rationale for the choice(s). If meta-analysis was performed, describe the model(s), method(s) to identify the presence and extent of statistical heterogeneity, and software package(s) used. |  |
|  | 13e | Describe any methods used to explore possible causes of heterogeneity among study results (e.g. subgroup analysis, meta-regression). |  |
|  | 13f | Describe any sensitivity analyses conducted to assess robustness of the synthesized results. |  |
| Reporting bias assessment | 14 | Describe any methods used to assess risk of bias due to missing results in a synthesis (arising from reporting biases). |  |
| Certainty assessment | 15 | Describe any methods used to assess certainty (or confidence) in the body of evidence for an outcome. |  |
| **RESULTS** | | |  |
| Study selection | 16a | Describe the results of the search and selection process, from the number of records identified in the search to the number of studies included in the review, ideally using a flow diagram. |  |
|  | 16b | Cite studies that might appear to meet the inclusion criteria, but which were excluded, and explain why they were excluded. |  |
| Study characteristics | 17 | Cite each included study and present its characteristics. |  |
| Risk of bias in studies | 18 | Present assessments of risk of bias for each included study. |  |
| Results of individual studies | 19 | For all outcomes, present, for each study: (a) summary statistics for each group (where appropriate) and (b) an effect estimate and its precision (e.g. confidence/credible interval), ideally using structured tables or plots. |  |
| Results of syntheses | 20a | For each synthesis, briefly summarise the characteristics and risk of bias among contributing studies. |  |
|  | 20b | Present results of all statistical syntheses conducted. If meta-analysis was done, present for each the summary estimate and its precision (e.g. confidence/credible interval) and measures of statistical heterogeneity. If comparing groups, describe the direction of the effect. |  |
|  | 20c | Present results of all investigations of possible causes of heterogeneity among study results. |  |
|  | 20d | Present results of all sensitivity analyses conducted to assess the robustness of the synthesized results. |  |
| Reporting biases | 21 | Present assessments of risk of bias due to missing results (arising from reporting biases) for each synthesis assessed. |  |
| Certainty of evidence | 22 | Present assessments of certainty (or confidence) in the body of evidence for each outcome assessed. |  |
| **DISCUSSION** | | |  |
| Discussion | 23a | Provide a general interpretation of the results in the context of other evidence. |  |
|  | 23b | Discuss any limitations of the evidence included in the review. |  |
|  | 23c | Discuss any limitations of the review processes used. |  |
|  | 23d | Discuss implications of the results for practice, policy, and future research. |  |
| **OTHER INFORMATION** | | |  |
| Registration and protocol | 24a | Provide registration information for the review, including register name and registration number, or state that the review was not registered. |  |
|  | 24b | Indicate where the review protocol can be accessed, or state that a protocol was not prepared. |  |
|  | 24c | Describe and explain any amendments to information provided at registration or in the protocol. |  |
| Support | 25 | Describe sources of financial or non-financial support for the review, and the role of the funders or sponsors in the review. |  |
| Competing interests | 26 | Declare any competing interests of review authors. |  |
| Availability of data, code and other materials | 27 | Report which of the following are publicly available and where they can be found: template data collection forms; data extracted from included studies; data used for all analyses; analytic code; any other materials used in the review. |  |

*From:*  Page MJ, McKenzie JE, Bossuyt PM, Boutron I, Hoffmann TC, Mulrow CD, et al. The PRISMA 2020 statement: an updated guideline for reporting systematic reviews. BMJ 2021;372:n71. doi: 10.1136/bmj.n71

For more information, visit: <http://www.prisma-statement.org/>

The eMERGe guidance are not available in checklist form, but can be found at the DOI below (France et al., 2019).

https://dx.doi.org/10.1186/s12874-018-0600-0

# **Appendix S2:** Example of the detailed search strategy used for OVID medline.

| **Concept** | **Subheading** | **Search Terms** |
| --- | --- | --- |
| ***Exposure –*** *neonatal mortality* | Infant mortality (exploded) | neonatal-mortality OR neonatal-death OR early-neonatal-death OR newborn-mortality OR newborn-death* OR new-born-mortality OR new-born-death* OR infant-mortality OR infant-death OR perinatal-mortality OR perinatal-death |
| ***Outcome –*** *experience of bereavement care* |  | experience-of-care or bereavement-care or care-after-neonatal-death or personal-experience or experience-of-bereavement-care or attitude or perspective or experience or opinion or view or interview or focus-group* or survey* or qualitative or questionnaire* |
| ***Situation Pt1 -*** *healthcare* | Hospitals (exploded) | healthcare-setting OR healthcare OR health-care OR hospital-setting OR hospital OR hospital-care OR clinical Or clinical-care OR clinical-setting OR facility OR facility-based |
| ***Situation Pt2 -*** *LMIC* |  | afghanistan[MeSH] OR albania[MeSH] OR algeria[MeSH] OR american samoa[MeSH] OR angola[MeSH] OR antigua and barbuda[MeSH] OR argentina[MeSH] OR armenia[MeSH] OR aruba[MeSH] OR azerbaijan[MeSH] OR bahrain[MeSH] OR bangladesh[MeSH] OR barbados[MeSH] OR republic of belarus[MeSH] OR belize[MeSH] OR benin[MeSH] OR bhutan[MeSH] OR bolivia[MeSH] OR bosnia and herzegovina[MeSH] OR botswana[MeSH] OR brazil[MeSH] OR bulgaria[MeSH] OR burkina faso[MeSH] OR burundi[MeSH] OR cabo verde[MeSH] OR cambodia[MeSH] OR cameroon[MeSH] OR central african republic[MeSH] OR chad[MeSH] OR chile[MeSH] OR china[MeSH] OR colombia[MeSH] OR comoros[MeSH] OR democratic republic of the congo[MeSH] OR congo[MeSH] OR costa rica[MeSH] OR cote d’ivoire[MeSH] OR croatia[MeSH] OR cuba[MeSH] OR cyprus[MeSH] OR czech republic[MeSH] OR djibouti[MeSH] OR dominica[MeSH] OR dominican republic[MeSH] OR ecuador[MeSH] OR egypt[MeSH] OR el salvador[MeSH] OR equatorial guinea[MeSH] OR eritrea[MeSH] OR estonia[MeSH] OR swaziland[MeSH] OR ethiopia[MeSH] OR fiji[MeSH] OR gabon[MeSH] OR gambia[MeSH] OR georgia (republic)[MeSH] OR ghana[MeSH] OR gibraltar[MeSH] OR greece[MeSH] OR grenada[MeSH] OR guam[MeSH] OR guatemala[MeSH] OR guinea[MeSH] OR guinea bissau[MeSH] OR guyana[MeSH] OR haiti[MeSH] OR honduras[MeSH] OR hungary[MeSH] OR india[MeSH] OR indonesia[MeSH] OR iran[MeSH] OR  iraq[MeSH] OR jamaica[MeSH] OR jordan[MeSH] OR kazakhstan[MeSH] OR kenya[MeSH] OR democratic people’s republic of korea[MeSH] OR republic of korea[MeSH] OR kosovo[MeSH] OR kyrgyzstan[MeSH] OR laos[MeSH] OR latvia[MeSH] OR lebanon[MeSH] OR lesotho[MeSH] OR liberia[MeSH] OR libya[MeSH] OR lithuania[MeSH] OR macau[MeSH] OR republic of north macedonia[MeSH] OR madagascar[MeSH] OR malawi[MeSH] OR malaysia[MeSH] OR indian ocean islands[MeSH] OR mali[MeSH] OR malta[MeSH] OR micronesia[MeSH] OR palau[MeSH] OR mauritania[MeSH] OR mauritius[MeSH] OR mexico[MeSH] OR moldova[MeSH] OR mongolia[MeSH] OR montenegro[MeSH] OR morocco[MeSH] OR mozambique[MeSH] OR myanmar[MeSH] OR namibia[MeSH] OR nepal[MeSH] OR netherlands antilles[MeSH] OR nicaragua[MeSH] OR niger[MeSH] OR nigeria[MeSH] OR oman[MeSH] OR pakistan[MeSH] OR panama[MeSH] OR papua new guinea[MeSH] OR paraguay[MeSH] OR peru[MeSH] OR philippines[MeSH] OR poland[MeSH] OR portugal[MeSH] OR puerto rico[MeSH] OR romania[MeSH] OR russia[MeSH] OR rwanda[MeSH] OR samoa[MeSH] OR sao tome and principe[MeSH] OR saudi arabia[MeSH] OR senegal[MeSH] OR serbia[MeSH] OR seychelles[MeSH] OR sierra leone[MeSH] OR slovakia[MeSH] OR slovenia[MeSH] OR melanesia[MeSH] OR somalia[MeSH] OR south africa[MeSH] OR south sudan[MeSH] OR sri lanka[MeSH] OR saint kitts and nevis[MeSH] OR saint lucia[MeSH] OR saint vincent and the grenadines[MeSH] OR sudan[MeSH] OR suriname[MeSH] OR syria[MeSH] OR tajikistan[MeSH] OR tanzania[MeSH] OR thailand[MeSH] OR timor leste[MeSH] OR togo[MeSH] OR tonga[MeSH] OR trinidad and tobago[MeSH] OR tunisia[MeSH] OR turkey[MeSH] OR turkmenistan[MeSH] OR uganda[MeSH] OR ukraine[MeSH] OR uruguay[MeSH] OR uzbekistan[MeSH] OR vanuatu[MeSH] OR venezuela[MeSH] OR vietnam[MeSH] OR middle east[MeSH] OR yemen[MeSH] OR yugoslavia[MeSH] OR zambia[MeSH] OR zimbabwe[MeSH] OR africa south of the sahara[MeSH] OR africa, central[MeSH] OR africa, northern[MeSH] OR africa, southern[MeSH] OR africa, eastern[MeSH] OR africa, western[MeSH] OR west indies[MeSH] OR indian ocean islands[MeSH] OR caribbean region[MeSH] OR central america[MeSH] OR latin america[MeSH] OR south america[MeSH] OR asia, central[MeSH] OR asia, northern[MeSH] OR asia, southeastern[MeSH] OR asia, western[MeSH] OR europe, eastern[MeSH] OR developing countries[MeSH] |

The search was limited to English language papers, with no date limits applied.

# **Appendix S3:** Table describing first order themes extracted from each study.

| **Reference** | **Theme 1 Emotional reaction** | **Theme 2**  **Social Relationships** | **Theme 3**  **Staff and Systems** | **Theme 4**  **Religion** | **Theme 5**  **Connection with the baby** | **Theme 6**  **Coping strategies** | **Theme 7**  **Economic** |
| --- | --- | --- | --- | --- | --- | --- | --- |
| **1** | Participants reported being confused by the diagnosis.  All mothers mentioned feeling lonely or empty. They reported feeling sad/distant, and some mentioned trying to be ‘brave’ and hide those feelings. Participants often grieved in private spaces but reported wanting somebody to talk to. Women reported feeling that they did things wrong in their pregnancy which caused the death (guilt). Women experiencing guilt reported anxiety about future pregnancies and wanted more reassurance.  Some women reported dreaming that their child was alive. | Mothers turned to other children and family for support. Husbands and parents were the major sources of support but close friends were also valued, and some reported receiving support from colleagues. Some mothers found full disclosure of their feelings challenging.  Generally, decisions about the burial and funeral were undertaken by the husband. | Mothers reported a lack of communication/ information/counselling, and some participants reported feeling that healthcare providers were trying to avoid talking to them. Participants wanted support on navigating grief, discharge, burial, etc…  Mothers also reported a lack of privacy, including being disturbed by other people, particularly other babies crying. | Many participants used religion to help cope with their loss. They reported praying and feeling relief through reading the Qur’an. One participant described the death as a test from Allah. | - | - | - |
| **16** | Nurses describe being personally affected by the death of neonates and reliance on colleagues in situations which are emotionally difficult.  Separation of professional attachment becomes easier over time and aids nurses coping, as they are aware that the situation is worse for the family.  If the Nurse feels they did everything possible for that baby and made the death as good as it could be then they may feel a sense of achievement. | Nurses reported that family were admitted as appropriate. This could be helpful and supported local customs for care of the neonate after death. | Strong bonds with families meant nurses felt they suffered more than other healthcare staff, particularly if they relate to the parents. They expressed concern surrounding how long to spend with each patient, particularly regarding quality of life.  Nurses valued psychologist support but also felt they needed more training to help mothers manage grief. Currently, nurses try to see if the mother wants comfort or privacy, and offer some degree of physical touch as comfort (eg: an arm/hand). Nurses reported feeling a sense of accomplishment if they are able to help the families, especially if the families return later to thank them. | Nurses reported that family often pray or baptise the baby when they visit. They also reported praying and asking God for comfort themselves following a death. | Nurses acknowledged the importance of allowing the mother time to connect with the baby after death. They reported closing the curtains to allow the mother to spend time with the baby, allowing silence as appropriate and offering opportunities to cuddle, bathe, change or clothe the baby. | Nurses discussed the unique difficulties associated with a death in a twin pregnancy, and how the surviving baby may help the mother cope with the loss. |  |
| **17** | Women described positive expectations turning to sorrow. Women who saw less of the nurse reported feelings of ‘abandonment’, and some women blamed the staff for their baby’s death. 12 women received no explanations for their baby’s death. Those who did receive an explanation found this positive.  Triggers for renewed grief included other people’s children. | One woman reported believing that her mother’s relation had cursed the pregnancy, and cited this as the reason for the death. | Women reported the physical presence and care of nurses as very positive aspects that indicated good care.  Some women reported feeling ignored and blamed the staff for their babies death, eg: attributing the death to inattentive nurses or nasal prongs for oxygen. Women reported wanting more interaction with the nurses and feelings of ‘abandonment’. One reported that a cleaner spoke to her following the death instead of a nurse.  Twelve women received no explanation for the death, and some reported finding it hard to evaluate care as they felt the nurses were more knowledgeable than them. | Some attributed the death to sins committed by other people such as an unfaithful husband or family. 5 women (1/4) mentioned the involvement of God. The use of God by healthcare professionals to rationalise the death was received positively. | - | - | - |
| **18** | Most women were unsure of the cause of their baby’s death and some women felt failed by the healthcare system.  This lack of understanding of what happened led some mothers to blame themselves.  Some women reported guilt associated with the death, which could lead to feelings of isolation. Some used family and healthcare workers as emotional support whilst others tried to hide their emotions and appear ‘strong’. Seeing other children was reported as a trigger for grief. | Two women reported receiving support from the community, and others reported using the family as emotional support. However, some women reported being blamed by family/community and accused of ‘carelessness’. | Most women were unsure of the cause of their baby’s death, and some women felt failed by the healthcare system as they hadn’t understood what was happening or felt their child had not been cared for appropriately.  Some used family and healthcare workers as emotional support, but healthcare providers were sometimes seen as uncaring, particularly when making the woman leave hospital immediately after the death. | Some reported that the death was ‘the will of God’. Religious practices were commonly used to cope with/rationalise the death. | Women who had been able to take care of their babies before they died were more likely to report waking at night or early morning. | - | - |
| **19** | Mothers described grief and a high level of concern for the unwell newborn. Generally feelings are described in negative tones but without specifics. These feelings could be isolating. | The community prioritised ‘useful’ older children and thus found neonatal mortality less concerning. In rural areas, it was said that the newborn “could be replaced by a new baby”. However, some parents disagreed with this and deaths of ‘precious’ children were considered more serious (eg: children who had been waited for or were only children). Newborn deaths were generally not spoken of and had far less celebration/ritual than if an elderly person died, as public mourning for a newborn was considered inappropriate.  For home/garden burials, one mother reported finding this constant reminded distressing. | Many babies were buried at the hospital.  Primary caregivers reported receiving little information on what was happening with their newborn.  Deaths of other children on the ward made the process more frightening. | Muslim babies were generally taken to cemeteries, whilst non-Muslim babies were buried outside the house or hospital. | - | - | Families were often forced to borrow money to fund hospital care, so spent the weeks after the death working extra or selling possessions to pay it back.  Iddir committees gave money after newborn death if the baby lived for a set time (range 24hrs to two months). |
| **20** | Mothers described a feeling of guilt as well as self-destructive thoughts and considerations of suicide.  Some reported feeling angry and a loss of control. They reported feeling that they weren’t responsible for their actions during this time.  Some participants reported fear surrounding future pregnancies and potential loss, and some noted differences in perceptions of pain felt between male/female partners were noted.  Some felt that they were the only one still grieving for their child in the longer term (eg: families, friends “forget”). Some reported fantasies about the child or feeling sensations of dying along with their baby. | There were some ambiguities reported with relationships with family and friends. While support was valued, some felt their feelings were downplayed or felt unable to express their grief. Women felt isolated in their grief in the longer-term and community attitudes centred around the grief being lessened by other children. Participants felt a need to give their child an identity and felt that the child lacked social recognition.  Participants also reported that their husbands experienced the pain differently and another woman reported her partner leaving after the death. One mentioned that their partner experienced a lack of support in his workplace. | Some women reported ineffectual support from healthcare professionals, focussing on the baby and ignoring the mother. Some noted that their male partner was ignored by staff even if the mother received support. | - | - | - | Economic support was reported as a key issue. |
| **21** | Mothers described the emotions associated with leaving the maternity ward, particularly the absence of the joy that should be felt, replaced with grief. This was also noted on receiving the news of the death and on arriving home without the baby. | Family/friends were described as supportive in general, with one participant describing a need to be with her siblings throughout the process. This was sometimes accompanied by a dismissive attitude and a perception of inadequate support, though participants acknowledge that this may have been designed to comfort the grieving parent. | Mothers reported lots of issues with the delivery of the news and of a lack of support/care from hospital staff. They comment on the content and form of the news being delivered, and disliked that they were not always told in person, and in one case a doctor entered the room to give medication unaware that the infant had died. | Mothers reported finding religion very helpful in managing the consequences of the death and it is reported that religion correlated with better psychological outcomes. | One mother reported wishing that she could go back to the moment of death and to have unwrapped/ bathed her child, asshe was not given the opportunity to do this at the time. | - | - |
| **22** | Mothers perceived learning about the loss as very hard and extremely painful. Some mothers expressed their emotions immediately whilst others exhibited delayed outward expressions of grieving, instead describing numbness, tiredness or emptiness on receiving the news. | The family were often over-protective of mothers and delayed breaking the news. In particular, they waited until a mother or mother-in-law was available to deliver it as this was considered ‘kinder’.  Preparation of the neonates body, bath and shrouding according to Islamic rituals was important, but often performed by family other than the mother even if she wanted to undertake it. | Hospital staff were reported to not speak to bereaved mothers and none of the mothers received counselling. | Mothers reported being told about the death through religious comforts such as the baby being in heaven. Mothers reported faith that God would ‘make it up to me’. One woman reported a three day funeral in which the baby was treated in the same way as an adult. | On receiving the news, some mothers immediately wished to connect by seeing the infant, holding belongings, etc.. Women were sometimes allowed to bathe and clothe the baby as time to grieve was considered important. However, no women obtained mementos like photos or footprints. Guilt, frustration and sadness for not connecting with the deceased infant prior and during the burial ceremony was common. Family often stopped the mother engaging with the baby and none of the mothers saw their baby buried. | - | - |
| **23** | At the moment of death, mothers reported feeling shocked, angry, guilty, regretful, and empty.  The experience of grief was reported to be recurring and could be bought on by triggers such as seeing other babies, mementos, sharing experiences and the lost child’s birthdays. Mothers reported emotional ‘ups and downs’ and stated that the memory of the baby would not be lost, but did note that grief diminished over time.  Some were also concerned about future pregnancies.  The presence of another child was reported to be helpful as well as a grief trigger. | The helpfulness of emotional support varied, in general, women reported more effective support from parents than husbands. | - | Many participants used God to cope with the loss of the baby. Women were advised to pray and remember Gods love. However, some women questioned religious beliefs following the death. | Participants reported that mementos could trigger grief. | Mothers reported adapting to the loss more than accepting it. Some rationalised the death as due to fate. Common coping strategies included turning to God, keeping busy with other children, taking pleasure from a new pregnancy or sharing stories. Some women also tried to suppress grief or reported that seeing other children was a grief trigger. | - |
| **24** | Fathers discussed the shock, devastation and pain of a happy moment becoming a sad one. They reported fear for the wellbeing of their partners, and reported feeling empty after the death. Some reported feeling weakness, helpless and discouraged. | Fathers described that being made to wait outside and not being by their partners sides was detrimental to their experience and that isolation increased their distress. They felt they were treated as outsiders and often got the news second/third hand, such as from cleaning staff/ janitors. Fathers felt that families/friends mainly considered the mothers grief. Many felt their role in supporting their wife was very important. | Uncertainty surrounding procedures and what would happen next were reported by fathers. | Fathers referred to the spiritual healing required and the spiritual presence of the baby. | Fathers reported that confronting the loss such as by holding the baby and arranging the funeral was helpful. Planning a new pregnancy was a positive experience once they had death with their loss. | Fathers’ reported that work activities and travel helped to distract them | - |
| **25** | Participants reported feeling pain, torn apart, stressed, helpless, weak and heartbroken following the death. Fathers' felt that they were responsible both for caring for the grieving mother and managing the funeral. | Close relatives and friends generally stayed in the house for 3 days following the bereavement and ceremonies followed were similar to those for an adult death rather than a miscarriage. Parents reported that the support was necessary and helpful. However, some women felt accused by partners/in-laws for the death, and some reported increased difficulties in their marriage. | Some participants blamed health care systems for the death, but some felt comforted/ consoled by the nurses caring for them. Systems such as no ambulances and poor roads also were reported as contributing to the death, which could lead participants to blame themselves and make the grief more challenging. | - | - | - | Parents reported feeling a loss of future wealth/  prestige with the death of a child. Fathers reported financial stresses and that having to continue to pay debts incurred by the pregnancy/ death was an additional concern. Some blamed lack of money and transport for the death. |
| **26** | Participants expressed grief, stress and fear surrounding the death of infants, and felt that these conflicted with their professional role. Many felt they had inadequate psychological preparation and did not know what to say/were afraid of making the situation worse. Many related what they had seen to movies and found this very distressing. | Midwifery students reported hiding their emotions or feeling unable to tell others about what they had experiences. They were also upset by how more senior midwives handled the infants without the care they expected. | Participants reported that they believed women experiencing a death should be delivered in a different area to others, and described being near to live infants as ‘cruel’. | - | - | - | - |

# **Appendix S4:** Detailed description of included studies.

| **Reference** | **Title** | **Location** | **World Bank Status** | **Facility Type** | **Sample Size** | **Sampling method** | **Data collection method** | **JBI score** | **Comments on risk of bias** |
| --- | --- | --- | --- | --- | --- | --- | --- | --- | --- |
| **1** | Psychosocial impact of perinatal loss among Muslim women. | Malaysia | Upper-middle-income country | Public and private | 16 (4 neonatal deaths, not possible to distinguish from other perinatal loss) | Purposive sampling | In-depth interviews and unstructured focus group. | 6 | Methodology lacks detail. Little reflexivity.  No standard reporting guidance used.  States data saturation reached. |
| **16** | Taking care of the newborn dying and their families: Nurses' experiences of neonatal intensive care. | Brazil | Upper-middle-income country | Private | 9 | Convenience sample | Semi-structured interviews | 7 | Methodology lacks detail. Little reflexivity.  No standard reporting guidance used. States data saturation reached. |
| **17** | Women's perceptions of Nurse-Midwives' caring behaviours during perinatal loss in Lilongwe, Malawi: an exploratory study. | Malawi | Low-income country | Community hospital | 20 | Purposive and snowball sampling. | Semi-structured interviews | 7 | Clear methodology with multiple researchers. Detailed reflexivity. No standard reporting guidance used. States data saturation reached. |
| **18** | Stillbirth, neonatal death and reproductive rights in Indonesia. | Indonesia | Lower-middle-income country | Public | 10 (2 neonatal deaths, not possible to distinguish from stillbirths) | Nested cohort study, non-random. | In-depth interviews | 6 | Methodology lacks detail. Little reflexivity.  No standard reporting guidance used. |
| **19** | What if the baby doesn't survive? Health-care decision making for ill newborns in Ethiopia. | Ethiopia | Low-income country | Public | 5 households | Purposive sampling. | Semi-structured in-depth interviews, focus groups and observation. | 8 | Clear methodology with two researchers. Some reflexivity. No standard reporting guidance used. States data saturation reached. |
| **20** | Understanding bereavement experiences of mothers facing the loss of newborn infants. | Brazil | Upper-middle-income country | Not stated | 6 | Purposive sampling. | Semi-structured interviews | 8 | Clear methodology with multiple researchers. Some reflexivity. Standard reporting guidelines (COREQ) used. States data saturation reached. |
| **21** | Communication of death and grief support to the women who have lost a newborn child. | Brazil | Upper-middle-income country | Public | 15 | Purposive sampling. | Semi-structured interviews | 7 | Moderately clear methodology, but no specifics on number of researchers. Little reflexivity. No standard reporting guidance used. |
| **22** | Informing mothers of neonatal death and the need for family-centred bereavement care: A phenomenological qualitative study. | Jordan | Upper-middle-income country | Public and private | 12 | Selected ‘with no specific order’. | Semi-structured interviews | 8 | Clear methodology with two researchers. Some reflexivity. Standard reporting guidelines (COREQ) used. States data saturation reached. |
| **23** | The experience of chronic sorrow among Indonesian mothers who have suffered recent perinatal loss. | Indonesia | Lower-middle-income country | Community health centres | 9 | Maximum variation sampling based on number of current children. | In-depth interviews and triangulation with family members. | 7 | Clear methodology with multiple researchers. Some reflexivity. No standard reporting guidance used. States data saturation reached. |
| **24** | Experience of Perinatal Death From the Father's Perspective. | Columbia | Upper-middle-income country | Private and public hospitals | 15 | Purposive and snowball sampling | In-depth, semi-structured interviews | 7 | Clear methodology, with only one researcher. Little reflexivity. No standard reporting guidance used. |
| **25** | "Your heart keeps bleeding": lived experiences of parents with a perinatal death in Northern Uganda. | Uganda | Low-income country | Hospitals & healthcare centres | 32 | Part of wider cluster, randomised trial. | Semi-structured in-depth interviews | 9 | Clear methodology with multiple researchers. Good reflexivity. No standard reporting guidelines used. States data saturation reached. |
| **26** | Experience of undergraduate midwifery students faced with perinatal death in clinical practice: A qualitative study. | China | Upper-middle income country | Tertiary hospitals | 12 | Purposive sampling | Semi-structured in-depth interviews | 7 | Clear methodology with multiple researchers. Some reflexivity. Standard reporting guidelines (COREQ) used. States that data saturation was reached. |
